# Functional Roles of Neural Preparatory Processes in a Cued Stroop Task Revealed by Linking Electrophysiology with Behavioral Performance

**DOI:** 10.1371/journal.pone.0134686

**Published:** 2015-07-31

**Authors:** Chao Wang, Mingzhou Ding, Benzi M. Kluger

**Affiliations:** 1 J Crayton Pruitt Family Department of Biomedical Engineering, University of Florida, Gainesville, Florida, United States of America; 2 Department of Neurology, University of Colorado Denver, Aurora, Colorado, United States of America; Zhejiang Key Laborotory for Research in Assesment of Cognitive Impairments, CHINA

## Abstract

It is well established that cuing facilitates behavioral performance and that different aspects of instructional cues evoke specific neural preparatory processes in cued task-switching paradigms. To deduce the functional role of these neural preparatory processes the majority of studies vary aspects of the experimental paradigm and describe how these variations alter markers of neural preparatory processes. Although these studies provide important insights, they also have notable limitations, particularly in terms of understanding the causal or functional relationship of neural markers to cognitive and behavioral processes. In this study, we sought to address these limitations and uncover the functional roles of neural processes by examining how variability in the amplitude of neural preparatory processes predicts behavioral performance to subsequent stimuli. To achieve this objective 16 young adults were recruited to perform a cued Stroop task while their brain activity was measured using high-density electroencephalography. Four temporally overlapping but functionally and topographically distinct cue-triggered event related potentials (ERPs) were identified: 1) A left-frontotemporal negativity (250-700 ms) that was positively associated with word-reading performance; 2) a midline-frontal negativity (450-800 ms) that was positively associated with color-naming and incongruent performance; 3) a left-frontal negativity (450-800 ms) that was positively associated with switch trial performance; and 4) a centroparietal positivity (450-800 ms) that was positively associated with performance for almost all trial types. These results suggest that at least four dissociable cognitive processes are evoked by instructional cues in the present task, including: 1) domain-specific task facilitation; 2) switch-specific task-set reconfiguration; 3) preparation for response conflict; and 4) proactive attentional control. Examining the relationship between ERPs and behavioral performance provides a functional link between neural markers and the cognitive processes they index.

## Introduction

Efficient goal-directed behavior requires neural mechanisms not only to react to immediate goal-related stimuli but also to proactively shift neural resources to optimize goal-directed behavior on the basis of environmental cues and internal goals. Cued task-switching paradigms reliably demonstrate that instructional cues impact both task performance and pre-stimulus brain activity, thus providing a relevant experimental model for studying the phenomenology and neural correlates of proactive cognitive control [1]. Despite several decades of research using these paradigms, controversy still exists regarding the precise cognitive processes triggered by instructional cues and their neural correlates [1,2].

To date, the majority of studies deduced the functional role of preparatory neural processes by measuring the effects of paradigm manipulations on behavior and neural markers. These studies report a diverse array of cue-related cognitive processes including goal-shifting [3], disengagement from the prior task [4], facilitation of task-relevant brain networks [5–7], inhibition of task-irrelevant brain networks [8], anticipatory conflict monitoring [9,10], implementation of anticipatory cognitive control for the more difficult task [11], and maintenance of goal representations [12,13]. These preparatory processes are temporally overlapping and may share neural resources making deductions based solely on paradigm manipulations problematic [14]. For example, in studies of cognitive control using the cued Stroop paradigm, a cue-related frontal slow wave around 400 ms or later and a parietal slow wave in the same latency range were frequently observed in the contrast between color-naming and word-reading cues [15,16]. However, it is unclear whether these components reflect facilitation of the task-relevant networks, inhibition of the task-irrelevant networks, preparation for response conflict, and/or general attentional control related with task difficulty. A notable limitation of traditional neuroimaging studies is the strong dependence on how cognitive constructs are operationalized and statistically tested with respect to task variations and contrasts. For example, regarding proactive cognitive control, it is common to examine only a single contrast between experimental conditions, such as between switch and repeat trials irrespective of task goals (e.g. [4,17–19]) or between different task goals irrespective of switching costs (e.g. [11,20–22]). In the case of the former it is known that switch and repeat trials differ not only in task-set reconfiguration but also in task difficulty, levels of task interference, and cue representation [23]. Thus preparatory activations revealed by comparing switch and repeat trials may include not only switch-specific preparatory processes but also activity related to general cognitive control and conflict monitoring that are present in both switch and repeat trials but relatively enhanced in switch trials. Notably, studies using multiple contrasts have overcome some of these limitations and have been successfully applied to resolve some apparent conflicts in this field [4].

To resolve these types of controversies, three complementary approaches have been proposed to obtain converging evidence to more directly assess the functional significance of neural markers, all of which make use of alterations in behavioral performance. First, task performance of patients with known lesions (e.g. stroke) or neurophysiologic aberrations (e.g. dopamine deficiency in Parkinson’s disease) can be compared to healthy control subjects [24]. Second, task performance may be compared before and after neurophysiologic manipulations (e.g. transcranial magnetic stimulation) [25]. Finally, studies may examine how variability in the amplitude or timing of a marker of neural activity (e.g. BOLD response, ERP, oscillatory activity) of interest predicts variations in behavioral responses across subjects or within subjects on a trial-to-trial basis [26–28]. For example, Coste et al. [29] using a cued Stroop task found that prestimulus fMRI activity in brain regions related to cognitive control (anterior cingulate cortex, dorsolateral prefrontal cortex; DLPFC), task-relevant sensory regions (e.g. color-sensitive area) and task-irrelevant areas (e.g. word-form area) predicted response time on a trial-by-trial basis for incongruent trials in subjects exhibiting a Stroop effect. Stern et al. [30] similarly found increased parietal and DLPFC activity with fMRI predicted response times for spatial trials on a spatial Stroop task. The main advantage of the third approach is that it takes advantage of naturally occurring neural and behavioral variability and does not require nervous system perturbation or recruitment of patients with specific lesions. To date, this approach has been successfully used to improve our understanding of the neurophysiology of basic perceptual, attentional and response time tasks but has rarely been used to understand more complex aspects of cognitive control [26–30].

In this study, we therefore sought to extend our knowledge of the functional role of cue-related neural preparatory processes by: (1) Identifying neural markers of preparatory processes using multiple contrasts from a single dataset; (2) Confirming the functional relevance of these markers by assessing whether variations in the amplitude of these markers were associated with intrasubject behavioral response variability; and (3) Examining patterns of behavioral facilitation across neural markers for dissociations suggestive of specific functions. We used a cued Stroop task and high-density electroencephalography to identify topographically distinct ERPs using both task-type contrasts (word-reading vs. color-naming) and transition-type contrasts (switch vs. repeat trials). Our specific study objectives were to: (1) Use task-type contrasts and correlations with task-performance to determine the function of task-related preparatory processes; (2) Use transition-type contrasts and correlations with task-performance to determine switch-related preparatory processes; and (3) Use correlations with task performance by stimulus attribute (congruent vs. incongruent) to determine conflict-related preparatory processes. By examining the overall pattern of behavioral results for each ERP obtained through these objectives we sought to deduce the specific functional role(s) of each ERP.

## Materials and Methods

### Participants

Sixteen college students, free from movement and neurological disorders and with normal or corrected-to-normal vision, participated in this study in exchange of course credits. All participants were right-handers and native English speakers. Two participants were excluded from analysis for (1) excessive motion artifacts and (2) poor connection between electrodes and scalp due to thick and dense hair. Data from 14 subjects (19.5 ± 3.9 years, 7 males) were included in the analyses reported here. All participants provided and signed written informed consent prior to participation. The research protocol was approved by the University of Florida Institutional Review Board.

### Study design and experimental paradigm

**Fig 1:**
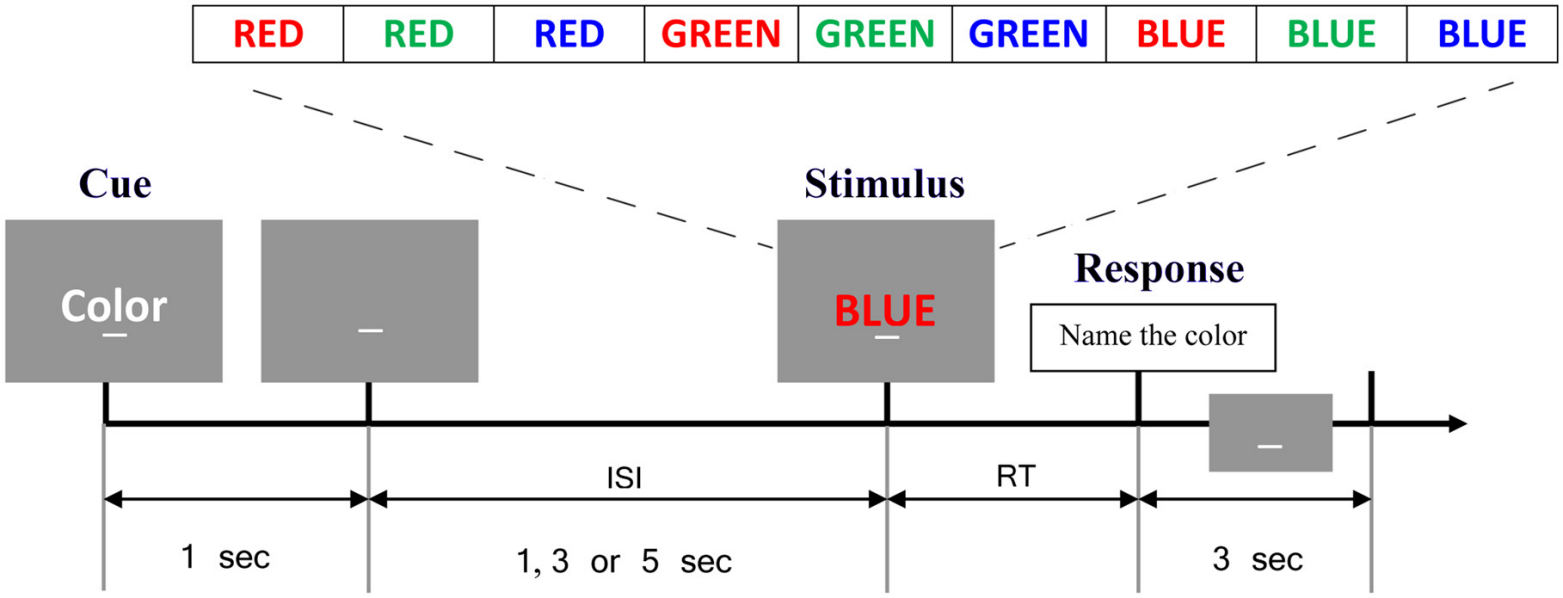
The Stroop task paradigm. In the beginning of each trial, an instructional cue consisting of either “word” for word-reading or “color” for color-naming was presented which was followed by a 1,3 or 5 second cue-target interval and an imperative color word stimulus (“red”, “blue” or “green”) written in colored letters. Any response would trigger a 3 second inter trial interval prior to the next cue. The time period of interest in this study was -500ms to 1500ms with 0ms indicating cue onset.

The experiment took place in an acoustically and magnetically shielded booth. All study objectives were pursued through distinct contrasts and analyses obtained from a single experimental recording of EEG and behavioral data described below. Subjects sat comfortably in a chair with their heads resting on a chin rest. Instructions and stimuli were presented on a LCD monitor placed approximately 85 cm in front of the chin rest using the Experimental Run Time System (Berisoft Corporation; Frankfurt, Germany). A computerized single-trial cued version of the Stroop task, originally developed by Cohen et al. [11], was performed by participants (see Fig 1). At the beginning of each trial, participants were presented with an instructional cue (“word” or “color”) lasting for 1 second. This was followed by a 1, 3 or 5 second cue-stimulus interval and an imperative color word stimulus (“red”, “blue” or “green”) written in colored letters. For the “word” task participants were instructed to read the word, while in the “color” task, participants were instructed to name the color of the letters. The “word” cue and the “color” cue were evenly divided, as were repeat and switch trials. On 60% of the trials the color of letters and the word were congruent (e.g. “red” written in red letters) and on the remaining 40% of trials the letters and word were incongruent (e.g. “red” written in green letters). Trial types were presented randomly in blocks of 120 balanced for cue type, congruency and cue-stimulus interval but not specifically balanced for switch type (repeat vs. switch). Subjects were asked to respond as quickly and accurately as possible. Reaction times (RTs) were determined by a microphone and voice activation software; the spoken words were manually recorded in real time by a research assistant. Participants received practice sessions of 30 trials to get familiarized with the task and to reduce learning effects. After practice, participants were asked to perform the task for forty minutes. The mean number of trials per subject was 274 (SD = 10).

### Electroencephalography (EEG) recording and processing

The EEG data was recorded using a 128-channel BioSemi Active Two System (Biosemi; Amsterdam, Netherlands) at a sampling rate of 1024 Hz. A CMS/DRL electrode loop served as ground and reference. The three-dimensional coordinates of each electrode, as well as three fiducial landmarks, were determined by means of a Polhemus spatial digitizer (Polhemus Inc.; Colchester, VT).

Off-line data preprocessing was performed using BESA 5.3 (BESA; Grafeling, Germany), EEGLAB [31] and custom scripts written in MATLAB 7.11 (Mathworks; Natick, MA). The continuous EEG data from each subject were first re-referenced to the average reference. The EEG signals were then band-pass filtered between 0.1 Hz and 83 Hz and downsampled to 250 Hz. The data within the period from -500 ms to 1500 ms relative to cue onset (0 ms) were epoched for the study of cue-related preparatory activity. Artifacts including eye movements and eye blinks, temporal muscle activity and line noise were removed from data epochs using the Infomax ICA algorithm implemented in EEGLAB [32]. After ICA, any epoch with incorrect behavioral response, abnormal RT (>3 s or <300 ms), and voltage exceeding 75 *μV* in any scalp channel, was rejected from further analysis. The total rejection rate of trials from each subject was between ~7% to ~30%.

Since the exact electrode locations are slightly different from subject to subject, spherical spline interpolation was applied to project each subject’s 128 channels of data into a standard 10–10 montage with 81 channels [33]. This procedure helps to improve the accuracy of group-level analysis. All of the following analyses were performed on the projected 81 channel data.

### Analysis of event related potentials (ERPs)

The ERP waveforms for each scalp electrode and task condition were extracted by averaging preprocessed epochs separately for each subject. The grand-average ERPs were low-pass filtered up to 30 Hz. A 200 ms period preceding the cue onset was used as baseline. Two contrasts of ERPs were used to study preparatory processes. First, we looked for task-type related preparatory processes by defining a contrast between color trials and word trials. Second, we defined a contrast between switch trials and repeat trials to reveal task-switch related processes.

The spatial distributions of the ERP contrasts were examined by topographic maps of ERP amplitude (the absolute value of ERP potential) differences for the selected time ranges. For task-type contrasts (word cue versus color cue), the topographic map was computed for the time range of 250–800 ms; a prior study by Perlstein et al. [15] using the same cued Stroop paradigm has identified a slow-wave difference between color and word cues around this time range. For transition type contrasts (switch trials versus repeat trials, the topographic map was computed for the time range of 450–800 ms; several prior studies [34–37] using cued task-switching paradigm have observed significant ERP difference between switch and repeat trials around this time range. The topographic maps were tested via electrode-by-electrode paired t-tests. The electrodes of interest were selected from scalp regions where two or more adjacent electrodes exhibited p < 0.05; these electrodes of interest were used for further ERP-RT association analysis.

### ERP-RT association analysis

To further investigate the functional significance of the identified ERP components from the condition contrasts, we examined the link between the ERP amplitudes and RTs. Trials of each task condition in the contrasts were sorted within subjects by RT into three equal-sized groups (slow, medium, and fast). The mean amplitudes of the identified ERP components were computed for each RT group and compared. For the association with RT by congruency conditions, the trials were divided into two groups (fast and slow) rather than three groups because of the small number of trials available for each condition.

### Statistical analysis

The behavioral data were tested via a 2 task type (color, word) × 2 congruency (congruent, incongruent) ×2 transition type (switch, repeat) repeated measures ANOVA. The sphericity assumption was checked by Mauchly’s test. For the selected electrodes the ERP waveform differences were tested through time via point-by-point paired t-tests. Type I error at α = 0.05 was controlled by the method described by Guthrie and Buchwald using an autocorrelation of 0.9 [38]. The differences of the ERP amplitudes from the different RT groups were tested via one-way repeated measures ANOVA. Paired t-tests were used for post hoc tests and the p-values were corrected for multiple comparisons by using the Holm-Bonferroni method [39]. P-values of less than 0.05 were considered statistically significant.

## Results

### Behavioral performance

The repeated measures ANOVA on the reaction time data revealed significant main effects of task type (F(1,13) = 7.85, p = 0.0150) and Stroop condition (F(1,13) = 34.91, p < 0.0001). RTs were generally slower for the color task than for the word task, and for the incongruent condition than for the congruent condition. The main effect of transition type was also significant (F(1,13) = 19.64, p = 0.0007). RTs for the task-switch condition were generally slower than for the task-repeat condition. There were no significant interactions between task type, congruency or transition type.

The repeated measures ANOVA on the accuracy data revealed significant main effects of congruency (F(1,13) = 28.59, p = 0.0001) and transition type (F(1,13) = 5.85, p = 0.0310). Participants made more errors for the incongruent condition and for the task-switch condition. The main effect of task type was not significant (F(1,13) = 2.27, p = 0.1559). There was a significant interaction between congruency and transition type (F(1,13) = 6.85, p = 0.0213). A larger congruency effect was observed in the task-switch condition. The interaction between congruency and task type showed a trend for significance (F(1,13) = 3.59, p = 0.0807) with larger congruency effect observed in the color task. No significant interaction was found between transition type and task type (F(1,13) = 0.02, p = 0.9016). The mean and standard deviation (SD) of RTs and accuracies for each condition are shown in Table 1.

**Table 1:** Stroop task performance by task type, transition type and congruency.

	Congruent		Incongruent	
	RT (ms)	Accuracy (%)	RT (ms)	Accuracy (%)
Color repeat	1064 (146)	99.81 (0.70)	1293 (232)	93.36 (8.55)
Color switch	1098 (160)	99.51 (0.98)	1304 (246)	90.47 (8.57)
Word repeat	1018 (153)	99.33 (1.92)	1177 (210)	98.10 (3.37)
Word switch	1055 (154)	99.48 (1.37)	1241 (179)	94.33 (7.39)

Standard deviations are shown in parenthesis.

### Study objective 1: Use task-type contrasts and correlations with task-performance to determine task-related preparatory processes

**Fig 2:**
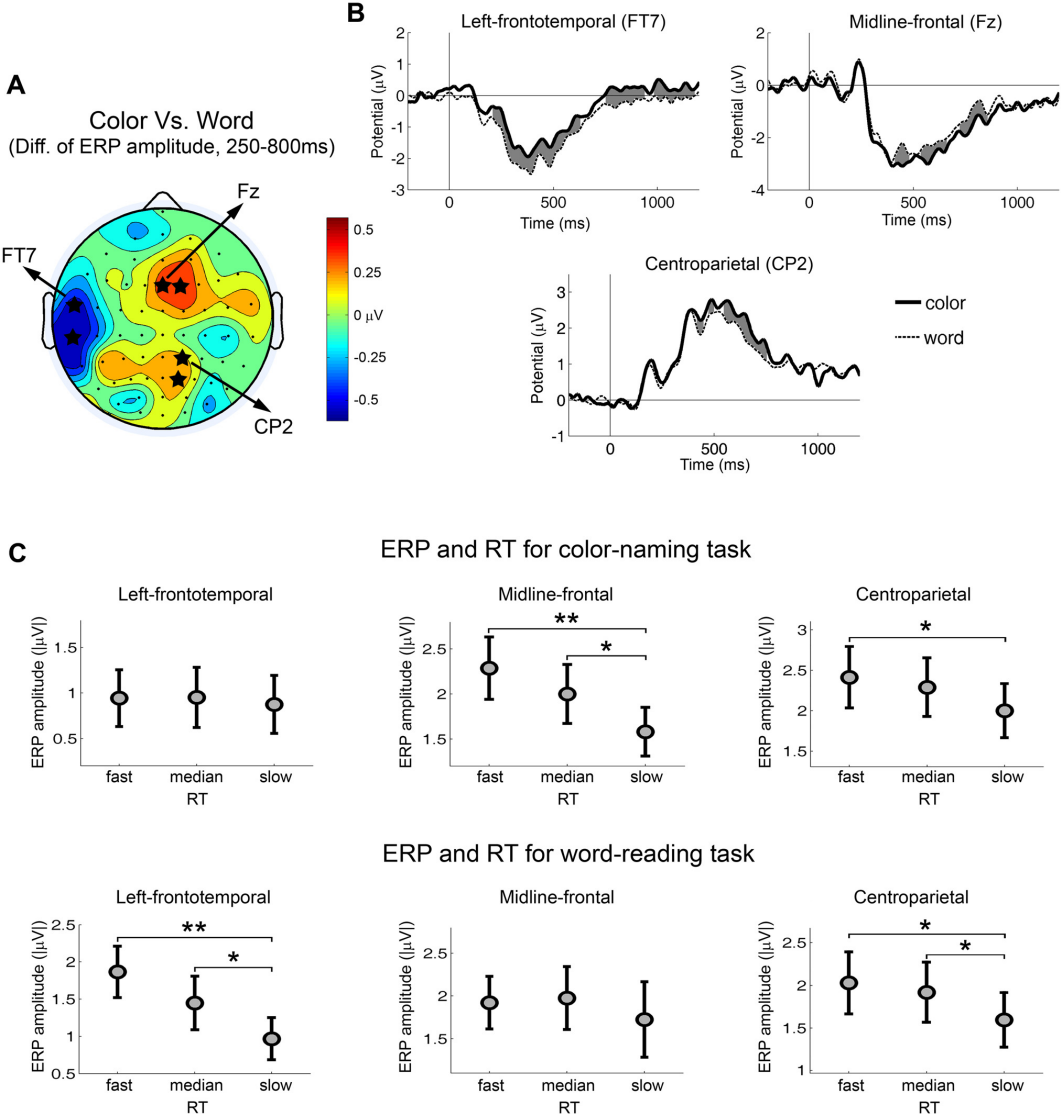
Task-related ERPs and their associations with reaction time. (A) Topography of the ERP difference from the contrast of color-naming versus word-reading trials. Blue color indicates larger ERPs for word-reading trials. Red color indicates larger ERPs for color-naming trials. The value that was used to compute the difference was the mean ERP amplitude within the period of 250–800 ms after the cue onset. The electrodes with significant ERP difference across subjects were marked by stars (*p* < 0.05). (B) Cue-evoked ERP waveforms from electrodes in the left-frontotemporal, the midline-frontal and the centroparietal scalp regions for color-naming trials and word-reading trials. Time zero is the cue onset. The time periods with significant ERP difference across subjects were shaded by gray color (*p* < 0.05). (C) ERP amplitudes for different RT groups by task type. Significant differences between RT groups were marked (** *p* < 0.01, * *p* < 0.05).

To determine the function of task-related preparatory processes we first identified such processes by comparing color versus word cues irrespective of transition type. Fig 2A shows the topographic map of the ERP amplitude difference within 250–800 ms between color and word cues. Three scalp regions were identified: 1) A left-frontotemporal region which showed higher ERP amplitude for the word-reading condition (most significant at FT7, p = 0.0009); 2) A midline-frontal region which showed higher ERP amplitude for the color-naming condition (most significant at Fz, p = 0.0107); and 3) A centroparietal region which showed higher ERP amplitude for the color-naming condition (most significant at CP2, p = 0.0085). The ERP waveforms from each scalp region were shown in Fig 2B. The time intervals with significant ERP difference are shaded in gray.

To understand the functional significance of these ERP components we next compared ERP amplitudes from different RT groups (i.e., trials with slow, medium, and fast RT) by task type. These analyses revealed three distinctive patterns (see Fig 2C): 1) for the left-frontotemporal negativity (250–700 ms), significant effect of RT group was found for word-reading task (F(2,26) = 5.53, p = 0.0100), but not for color-naming task (F(2,26) = 0.10, p = 0.9023). Fast RT group was linked to larger ERP amplitude in word-reading trials (fast > slow, p = 0.0036; median > slow, p = 0.0463); 2) for the midline-frontal negativity (450–800 ms), significant effect of RT group was found for color-naming task (F(2,26) = 4.35, p = 0.0235), but not for word-reading task (F(2,26) = 0.43, p = 0.6534). Fast RT group was linked to larger ERP amplitude in color-naming trials (fast > slow, p = 0.0089; median > slow, p = 0.0494); and 3) for the centroparietal positivity (450–800 ms), the effect of RT group was significant for both color-naming (F(2,26) = 4.29, p = 0.0245) and word-reading tasks (F(2,26) = 6.39, p = 0.0055). Fast RT group was linked to larger ERP amplitude in color-naming trials (fast > slow, p = 0.0336) as well as in word-reading trials (fast > slow, p = 0.0124; median > slow, p = 0.0326).

### Study objective 2: Use transition-type contrasts and correlations with task-performance to determine switch-related preparatory processes

**Fig 3:**
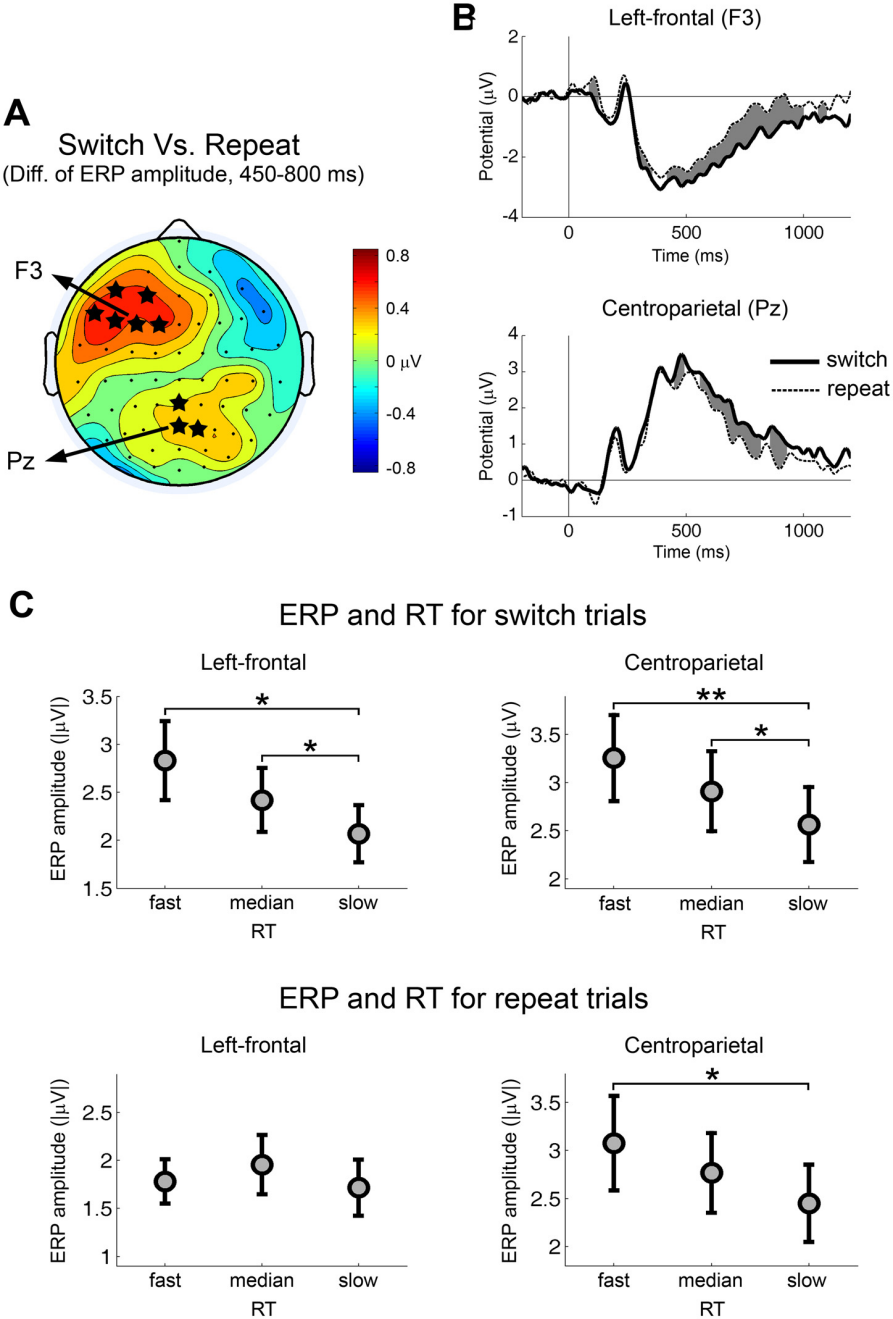
Switch-related ERPs and their association with reaction time. (A) Topography of the ERP difference from the contrast of task-switch versus task-repeat trials. Red color indicates larger ERPs for switch trials. Blue color indicates larger ERPs for repeat trials. The value used to compute the difference was the mean ERP amplitude within the period of 450–800 ms after the cue onset. The difference was computed for each subject and the grand-average was plotted in the figure. The electrodes with significant ERP difference across subjects were marked by stars (*p* < 0.05). (B) Cue-evoked ERP waveforms from electrodes in the left-frontal and the centroparietal scalp regions for task-switch trials and task-repeat trials. Time zero is the cue onset. The time periods with significant ERP difference were shaded by gray color (*p* < 0.05). (C) ERP amplitudes for different RT groups by transition type. Significant differences between RT groups were marked (** *p* < 0.01, * *p* < 0.05).

To determine the function of task-switch-related preparatory processes we first identified such processes by comparing switch versus repeat trials irrespective of task type. Fig 3A shows the topographic map of the ERP amplitude difference within 450–800 ms between switch trials and repeat trials. This analysis identified two scalp regions: 1) A left-frontal region which showed larger ERP amplitudes for switch trials (most significant at F3, p = 0.0067); and 2) A centroparietal region which showed larger ERP amplitude for switch trials (most significant at Pz, p = 0.013). The ERP waveforms from each scalp region were shown in Fig 3B. The time intervals with significant ERP difference are shaded in gray.

To better understand the functional significance of these ERP components we next compared ERP amplitudes from different RT groups by transition type. These analyses revealed (see Fig 3C): 1) for the left-frontal negativity (450–800 ms), significant effect of RT group was found for switch trials (F(2,26) = 4.75, p = 0.0175), but not for repeat trials (F(2,26) = 0.58, p = 0.5644). Fast RT group was linked to larger ERP amplitude in switch trials (fast > slow, p = 0.0146; median > slow, p = 0.0458); and 2) for the centroparietal positivity (450–800 ms), the effect of RT group was significant for both switch (F(2,26) = 6.95, p = 0.0038) and repeat trials (F(2,26) = 3.92, p = 0.0324). Fast RT group was linked to larger ERP amplitude in switch trials (fast > slow, p = 0.0055; median > slow, p = 0.0315) as well as in repeat trials (fast > slow, p = 0.0486).

### Study objective 3: Use correlations with task performance by stimulus attributes (congruent vs. incongruent) to determine conflict-related preparatory processes

**Fig 4:**
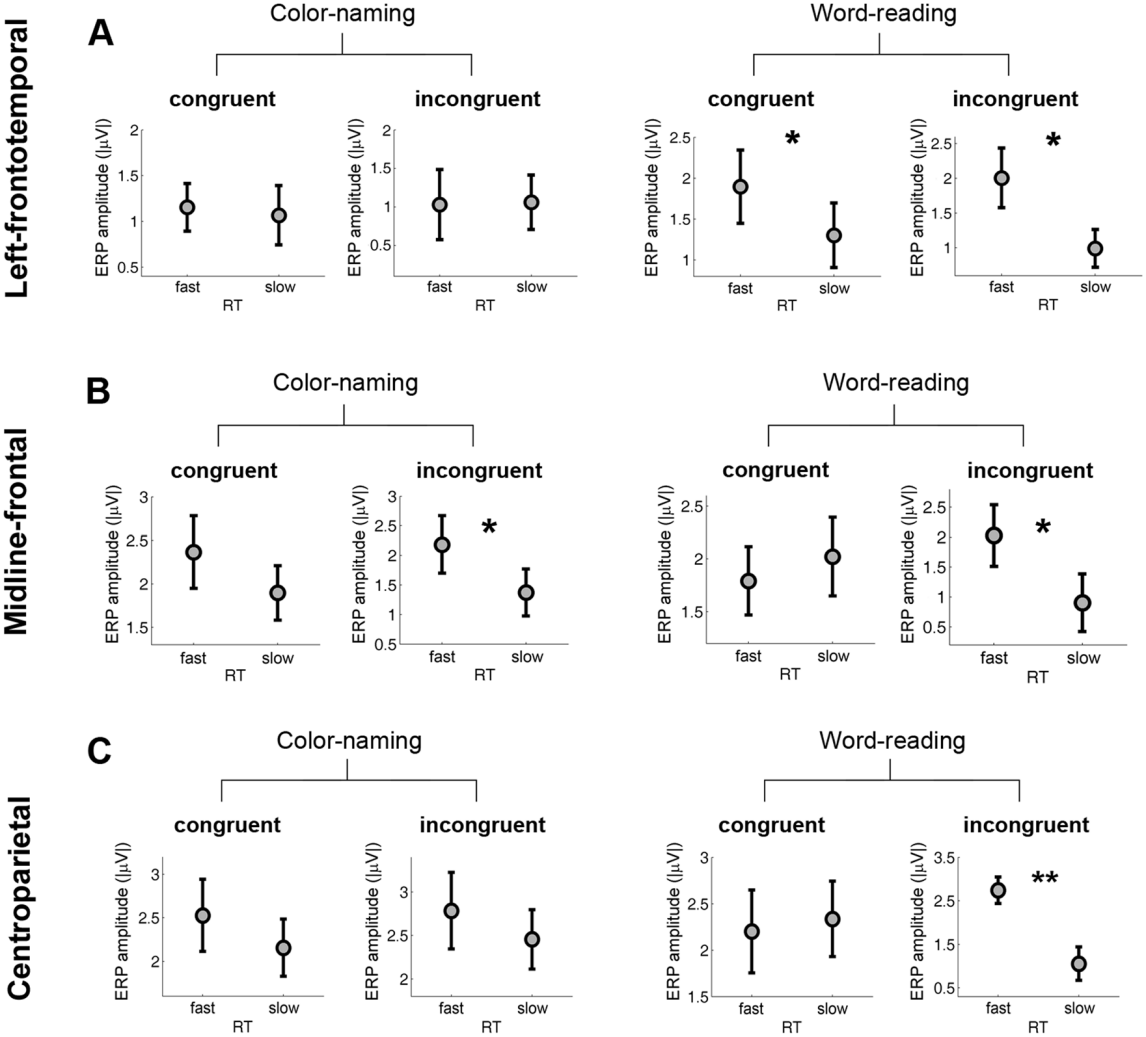
Task-related ERPs and their association with RT by congruency. ERP amplitudes are plotted for different RT groups by task and stimulus type (congruous vs. incongruous) for (A) the left-frontotemporal negativity (FT7, 250–700 ms), (B) the midline-frontal negativity (Fz, 450–800 ms), and (C) the centroparietal positivity (CP2, 450–800 ms). Significant differences of ERP amplitudes between RT groups were marked (** *p* < 0.01, * *p* < 0.05). Because of the small number of trials available for analysis two RT groups (fast and slow) instead of three RT groups were used.

To better understand the functional roles of ERP components identified through task contrasts under Study objective 1 we compared ERP amplitudes by RT groups for congruent and incongruent stimuli within each task type (see Fig 4). These analyses revealed: 1) for the left- frontotemporal negativity (250–700 ms), fast RT group was linked to larger ERP amplitude in word-reading trials regardless of congruency (congruent: fast > slow, p = 0.0384; incongruent: fast > slow, p = 0.0474); 2) for the midline-frontal negativity (450–800 ms), fast RT group was linked to larger ERP amplitude in incongruent trials regardless of task type (color-naming: fast > slow, p = 0.0440; word-reading: fast > slow, p = 0.0396); and 3) for the centroparietal positivity (450–800 ms), fast RT group was linked to larger ERP amplitude only in word-reading incongruent trials (fast > slow, p = 0.0082).

**Fig 5:**
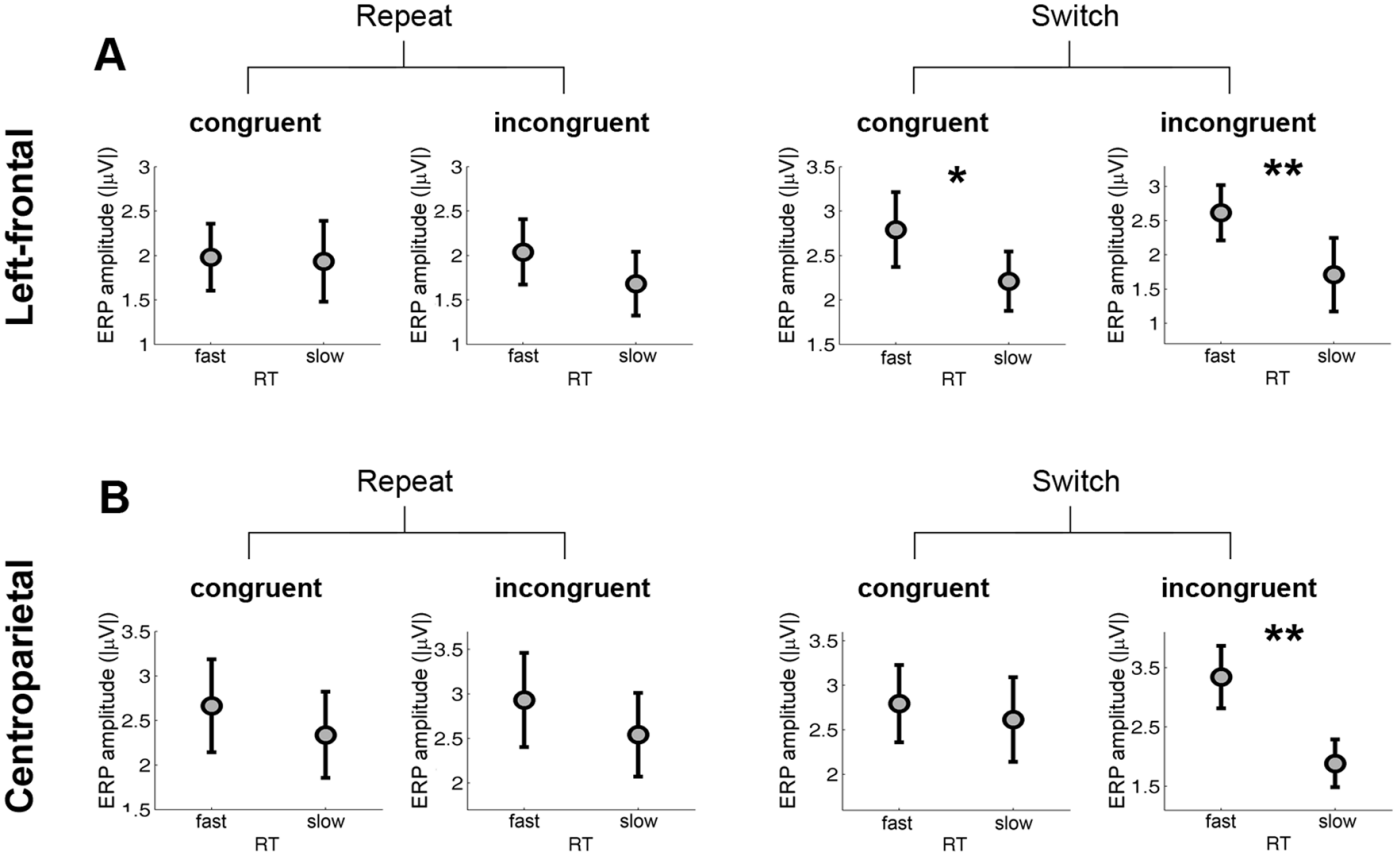
Switch-related ERPs and their association with RT by congruency. ERP amplitudes are presented for different RT groups by switch and stimulus type (congruous vs incongruous) for (A) the left-frontal negativity (F3, 450–800 ms), and (B) the centroparietal positivity (Pz, 450–800 ms). Significant differences of ERP amplitudes between RT groups were marked (** *p* < 0.01, * *p* < 0.05). Because of the small number of trials available for analysis two RT groups (fast and slow) instead of three RT groups were used.

To better understand the functional roles of ERP components identified through switch contrasts under Study objective 2 we compared ERP amplitudes by RT groups for congruent and incongruent stimuli for switch and repeat trials (see Fig 5). These analyses revealed: 1) for the left-frontal negativity (450–800 ms), fast RT group was linked to larger ERP amplitude in switch trials regardless of congruency (congruent: fast > slow, p = 0.0215; incongruent: fast > slow, p = 0.0078); and 2) for the centroparietal positivity (450–800 ms), fast RT group showed larger ERP amplitude than slow RT group only in the switch incongruent condition (p = 0.0018). For Figs 4 and 5, because the number of trials available for analysis was small, only two RT groups (fast and slow) were used.

### Summary of Results: Examine the overall pattern of behavioral results for each ERP component to deduce their functional roles

Table 2 summarizes the results of the prior three Study objectives and shows that each topographically distinct ERP component has a unique pattern of behavioral performance facilitation. The left-frontal negativity facilitated switch trials irrespective of task type or congruency, and is consistent with a primary role of task-set reconfiguration. The left-frontotemporal negativity facilitated word-reading trials irrespective of transition type or congruency, and is consistent with a primary role of engaging task-specific (language/reading) resources. The midline-frontal negativity facilitated the color naming task as well as incongruent stimuli for both task types, and is consistent with a primary role of proactively engaging attentional resources for stimuli with high potential conflict or difficulty. The centroparietal positivity facilitated both task types and both transition types, and is consistent with a primary role for facilitating general proactive cognitive control.

**Table 2:** ERP components, their correlations with behavioral performance, and their proposed functional roles.

ERP components	Correlation with RT			Proposed function
	Task type	Transition type	Congruency	
Left-frontal negativity (450–800 ms)	None	Switch	None	Task-set reconfiguration
Left-frontotemporal negativity (250–700 ms)	Word	None	None	Task specific (language) preparation
Midline-frontal negativity (450–800 ms)	Color	None	Incongruent	Anticipation of response conflict
Centroparietal positivity (450–800 ms)	Color and word	Switch and repeat	Incongruent	General proactive attentional control

## Discussion

Supporting our initial objective, we found that the functional significance of neural preparatory processes could be deduced by examining the pattern of their relationship to behavioral performance. Specifically, our data implicated four topographically distinct and functionally dissociable ERPs to serve unique behavioral roles: 1) domain-specific task facilitation (left-frontotemporal negativity); 2) switch-specific task-set reconfiguration (left-frontal negativity); 3) preparation for high conflict stimuli (midline-frontal negativity); and 4) proactive attentional control (centroparietal positivity). As discussed below, these results are consistent with several prior studies in this area, most of which typically focused on the role of only one or two of these components [3,13,35]. This congruence with prior research suggests that this approach may be a valuable source of converging evidence complementing traditional neuroimaging studies in healthy adults and can provide empiric support for the existence of hypothesized cognitive processes as well as uncover novel underlying mechanisms. These results further suggest that explanations of preparatory neural activity may need to more broadly include multiple functions and that approaches viewing these functions as mutually exclusive may be misdirected.

Of note, while differences in the topographic distribution of ERPs in the context of this study can be safely assumed to reflect differences in neural sources among the ERPs. However, scalp topography does not necessarily indicate the specific brain areas generating the ERPs as the depth, orientation, and location of neuronal populations generating an ERP all affect scalp topography.

### Task-specific facilitation

Left-frontotemporal negativity amplitude during 250–700 ms was specifically associated with faster word-reading performance and did not appear to be associated with congruence or switch status. These findings strongly suggest that this ERP component is a marker for a word-reading specific preparatory process which enhanced task performance via amplification of the task-relevant brain network. Although our data does not allow for adequate spatial resolution to determine its precise neural origin, we hypothesize that it represents the activity of the left perisylvian language structures which are known to be more involved in reading (e.g. pars triangularis) than speech production [40]. However, it is also possible that activation of more generic speech generation areas facilitate the more automatic word-reading task but are not sufficient by themselves to improve color-naming performance. Notably, there did not appear to be any inverse associations between ERP activity and performance to suggest the active inhibition of task-irrelevant activity. While such inhibition may occur, it probably does so more on the stimulus and response levels than as a proactive process [41]. These results lend support to prior studies which suggest that priming of task-relevant areas is one important function indexed by cue-induced activity [5,6].

### Task-set reconfiguration

Regarding task-switching, left-frontal negativity amplitude during 450–800 ms was specifically associated with switch trial performance, and did not appear to be associated with task type or congruency. These findings strongly suggest that this ERP component is a marker for switch-specific task-set reconfiguration processes such as disengagement from the prior task and engagement of the required task [42–44]. The lack of ERP-performance association for repeat trials suggests that this ERP component reflects a switch-only preparatory process rather than a preparatory process that is present in both switch and repeat trials but has a stronger activation in switch trials [45]. This lends support to the two-stage models of task switching proposed by Rogers et al. [46], Rubinstein et al. [3] and Meiran [47] where switch-specific processes of task-set reconfiguration are presumed to exist and are dissociable from other task-related processes. The scalp distribution of this ERP component is consistent with prior lesion studies which reported that damage to left prefrontal cortex but not right prefrontal cortex led to impaired task-switching performance [48,49]. The centroparietal positivity amplitude was also associated with task-switching on our task and have been associated with task-switching in other studies [35,37,50,51]. Notably, our results showed that the centroparietal positivity was associated with both switch and repeat performance but more engaged for switch trials suggesting that it is serving a more general function in task preparation. Piguet et al. [15] found a similar pattern in their fMRI study and also concluded that this parietal process serves a more general function as will be discussed below.

### Preparation for response conflict

The midline-frontal negativity amplitude during 450–800 ms showed associations with color-naming performance and was also associated with performance on incongruent stimuli for both word reading and color naming tasks. This pattern of results suggests that this ERP component is likely the index of neural preparatory processes elicited by the anticipation of high conflict stimuli. The reason is that both color-naming and the processing of incongruous stimuli represent high-conflict within this paradigm. The midline-frontal distribution of this ERP component would suggest an origin in the medial frontal structures, including anterior cingulate cortex (ACC), supplementary motor areas (SMA), pre-SMA and other spatially proximal regions. The classic conflict monitoring model [52,53] proposes that ACC detects conflicts during the action selection process and signals the need for increased top-down control. Although this model was originally formulated for reactive control, several recent studies have suggested that it could be extended to proactive control of anticipated conflict [9,10]. Specifically, Sohn et al. [10] performed an fMRI study and reported higher ACC responses for high versus low conflict cues, indicating a role of ACC in conflict anticipation.

### General proactive attentional control

The amplitude of the centroparietal positivity during 450–800 ms showed a complex and broad set of positive associations with performance in a variety of conditions, including word-reading and color-naming tasks, switch and repeat trials, incongruent stimuli for the word reading, and switch trials. Interestingly, this ERP component did not significantly associate with RT of incongruent trials in color-naming task, which is the condition that presumably requires the most cognitive control. This pattern of results suggests that the centroparietal positivity is a marker for more general proactive attentional control [44] instead of for a control process that is more specific to anticipation of response conflict. The posterior positivity with a latency around 400 ms or more has been consistently reported during task preparation as a correlate of task-switching [35,37,50,51]. In contrast with our finding, Karayanidis et al. [51] reported that the switch-related centroparietal positivity was linked to RT for switch but not repeat trials. It is worth noting that in their paradigm a far briefer cue-to-stimulus interval (600 ms) was used which may leave little time for neural preparatory processes to fully develop. Most likely our identified centroparietal positivity consists of multiple temporally overlapped subcomponents with at least one of them specifically linked with task-switching [34,54]. These subcomponents may originate from multiple distinct but spatially proximate areas. Further dissociation could be studied by source localization analysis.

### Summary, limitations and outlook

Overall the findings reported here provide evidence that several parallel neural preparatory processes were evoked by the instructional cues, including task-specific facilitation, task-set reconfiguration, anticipation of conflict and general proactive attention, and that these neural preparatory processes are indexed by distinct ERP markers. Associating ERPs with behavioral performance proves to be a key step linking these ERP neural markers to specific cognitive operations. Limitations of the study include a relatively small sample size, use of a single task, and the limited spatial resolution of EEG for determining the active neural sources of observed ERP activity. Future research, including combined fMRI/ERP studies, the use of multiple contrasts, and comparison across tasks is suggested to further clarify these results.
